# Questioning the Quality of 16S rRNA Gene Sequences Derived From Human Gut Metagenome-Assembled Genomes

**DOI:** 10.3389/fmicb.2021.822301

**Published:** 2022-02-04

**Authors:** Pranvera Hiseni, Lars Snipen, Robert C. Wilson, Kari Furu, Knut Rudi

**Affiliations:** ^1^Department of Chemistry, Biotechnology and Food Sciences, Norwegian University of Life Sciences, Ås, Norway; ^2^Genetic Analysis AS, Oslo, Norway; ^3^Department of Biotechnology, Faculty of Applied Ecology, Agricultural Sciences and Biotechnology, Inland Norway University of Applied Sciences, Hamar, Norway

**Keywords:** 16S rRNA, metagenome assembled genome (MAG), metagenome analyses, human gut microbiome, prokaryotic genome

The recent introduction of metagenome-assembled genomes (MAGs) has marked a major milestone in the human gut microbiome field (Almeida et al., 2019; Nayfach et al., 2019; Pasolli et al., 2019). Such reference-free, *de novo*-assembled genomes (Hugerth et al., 2015) have revealed a wide range of hitherto uncultured microbial species in human gut samples.

The significance of MAGs in unraveling human gut microbial diversity was supported by their overwhelming representation in a comprehensive human gut prokaryotic collection filtered by metagenome data dereplicated at 97.5% average nucleotide identity (ANI) (Hiseni et al., 2021). More than 90% of the collection consists of MAGs, while the rest of the collection mainly comprises RefSeq genomes (Figure 1A).

**Figure 1:**
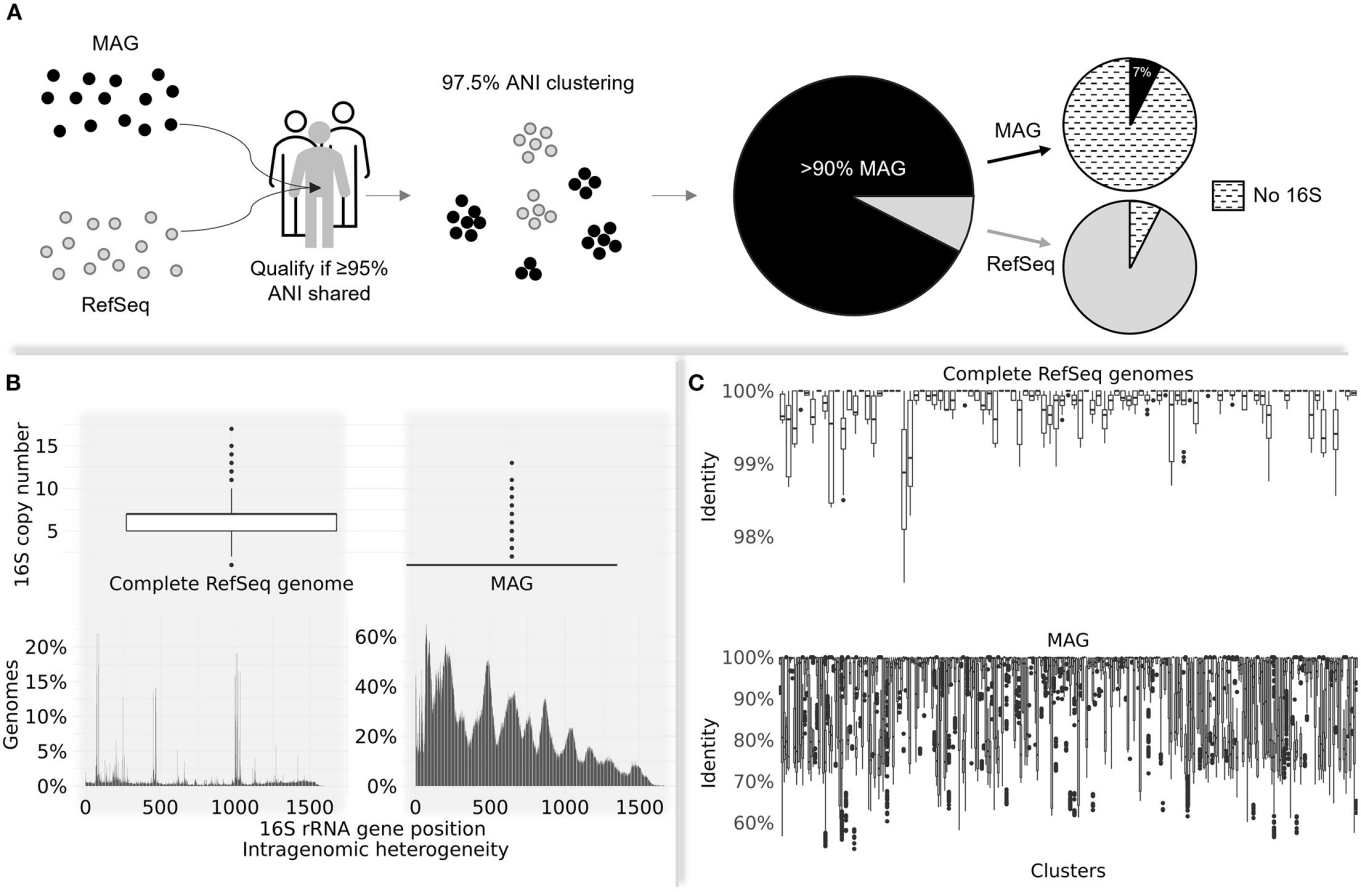
**(A)** The process of filtering human gut-derived MAGs and RefSeq prokaryotic genomes against a pool of >3,500 non-redundant healthy human gut metagenomes. Only genomes sharing ≥95% average nucleotide identity (ANI)—a conventional threshold marking species delineation (Jain et al., 2018)—were kept for further processing. The qualified genomes dereplicated at 97.5% ANI were mostly represented by MAGs (>90%). Only 7% of MAGs harbored detectable 16S rRNA gene sequences, while the opposite was observed in RefSeq genomes (7% lacked detectable 16S). **(B)** The distribution of 16S copy numbers on complete RefSeq genomes vs. MAGs (upper panel); the intragenomic 16S rRNA gene heterogeneity on genomes with multiple 16S copies for the same groups (bottom panel). MAGs are associated with increased intragenomic variability across all positions compared to RefSeq genomes. **(C)** The average nucleotide identity of 16S sequences belonging to the same 97.5% ANI cluster. Each boxplot refers to one cluster. The upper panel depicts clusters made of pure complete RefSeq genomes, while the bottom panel shows the distribution of shared identities on clusters entirely comprising MAGs. RefSeq-derived 16S sequences within same clusters show high identity (average of 99.8%); MAG clusters contain highly variable 16S sequences, with an average identity of 93%.

A great challenge related to MAGs is their lack of 16S rRNA sequences. Skewed species abundance, high 16S sequence similarity, and high volumes of short-reads data cause major difficulties for assembling the sequences of this gene (Yuan et al., 2015), frequently rendering these genomes incomplete.

A barrnap search (https://github.com/tseemann/barrnap) revealed that from >270,000 qualified MAGs, only 7% yielded 16S sequences, while this gene was found in 93% of >106,000 other genome types. MAGs positive for 16S had a significantly lower copy number compared to complete RefSeq genomes (Figure 1B; top panel) and substantially higher intragenomic variance (Figure 1B; bottom panel). Challenges in obtaining multiple 16S copies from incomplete genomes are well-described in the literature (Perisin et al., 2016; Louca et al., 2018); however, to exacerbate the problem, their enormous intragenomic heterogeneity renders their overall quality questionable.

A multiple sequence alignment of 16S rDNA sequences extracted from members of identical 97.5% ANI clusters, followed by the computation of their distance [*ape* package in RStudio (Paradis and Schliep, 2018)], has revealed that clusters consisting purely of MAGs share on average 93% identity, as contrasted by 99.8% average 16S sequence identity in clusters made of pure, complete RefSeq genomes (Figure 1C).

Considering that 16S is a highly conserved gene, its identity among same-cluster genomes was expected to be higher than the threshold used for dereplicating them (>97.5%; Kim et al., 2014; Jain et al., 2018). The excessive 16S divergence among MAG-only clusters raises red flags, potentially reflecting issues related to their assembly, as previously reported (Nelson et al., 2020; Meziti et al., 2021).

All MAGs studied here were >95% complete with <5% contamination, a conventional criterion marking their high quality. Given the extreme importance of the 16S gene in microbial taxonomy and ecology, it seems unacceptable that MAGs can be labeled as such and at the same time contain low-quality information about this single most important gene that links the re-constructed genomes to the huge body of 16S-based microbiota studies conducted worldwide.

Furthermore, the acceptance of poor 16S rDNA quality in MAGs currently excludes a majority in the microbial research community that does not have the economic or computational resources to perform large-scale shotgun sequencing.

## Author Contributions

KR and PH conceived the idea. PH wrote the manuscript with an equal input from all authors. All authors discussed and interpreted the findings. All authors contributed to the article and approved the submitted version.

## Funding

This work was financially supported by Norway Research Council, a Norwegian government agency funding research and innovation, through R&D project grant nos. 283783, 248792, and 301364.

## Conflict of Interest

PH and KF were employed by company Genetic Analysis AS. The remaining authors declare that the research was conducted in the absence of any commercial or financial relationships that could be construed as a potential conflict of interest.

## Publisher's Note

All claims expressed in this article are solely those of the authors and do not necessarily represent those of their affiliated organizations, or those of the publisher, the editors and the reviewers. Any product that may be evaluated in this article, or claim that may be made by its manufacturer, is not guaranteed or endorsed by the publisher.
